# Intensive vs standard blood pressure control in adults with type 2 diabetes: a systematic review and GRADE-based meta-analysis

**DOI:** 10.1097/MS9.0000000000004879

**Published:** 2026-04-07

**Authors:** Zaryab Bacha, Javeria Javed, Shree Rath, Maheen Sheraz, Umama Alam, Ehtesham Wali Khan, Sufyan Shahid, Sajjad Ghanim Al-Badri, Nirmal Noor, Fathimathul Henna, Fazia Khattak, Fatima Sajjad, Iqra Shahid, Ahmad Khan, Saqib Hayat Khan, Mamur Khan, Kamil Ahmad Kamil

**Affiliations:** a Department of Medicine, Khyber Medical College, Peshawar, Pakistan; b Department of Medicine, Jinnah Sindh Medical University, Karachi, Pakistan; c Department of Medicine, All India Institute of Medical Sciences, Bhubaneswar, India; d Department of Medicine, Continental Medical College, Lahore, Pakistan; e Department of Medicine, Khawaja Muhammad Safdar Medical College, Sialkot, Pakistan; f College of Medicine, University of Warith Al-Anbiyaa, Karbala, Iraq; g Department of Medicine, People’s University of Medical and Health Sciences for Women, Nawabshah, Pakistan; h Department of Medicine, Dubai Medical College for Girls, Dubai, UAE; i Department of Medicine, King Edward Medical University, Lahore, Pakistan; j Internal Medicine Department, Mirwais Regional Hospital, Kandahar, Afghanistan

**Keywords:** hypertension, meta analysis, older adults, standard blood pressure control, type 2 diabetes mellitus

## Abstract

**Background:**

The optimal blood pressure (BP) target for older adults with type 2 diabetes (T2DM) remains controversial. This meta-analysis compares intensive BP control (IBPC) versus standard BP control in T2DM patients.

**Methods:**

Following PRISMA guidelines, we searched PubMed, Embase, and Cochrane Central through March 2025 for randomized controlled trials (RCTs) comparing IBPC (target <130/80 mm Hg) with standard control (<140/90 mm Hg) in adults ≥35 years with T2DM. Primary outcome was a composite of nonfatal stroke, nonfatal myocardial infarction, cardiovascular (CV) death, or heart failure hospitalization. Secondary outcomes included microvascular complications and safety. Data were pooled using random-effects models (*I*^2^ > 50%) or fixed-effects models (*I*^2^ ≤ 50%).

**Results:**

Ten RCTs (*n* = 23 826) were included. IBPC significantly reduced the primary composite outcome (OR: 0.82, 95% CI: 0.74–0.91, *P* = 0.0001, *I*^2^ = 0%) and stroke risk (OR: 0.62, 95% CI: 0.49–0.79, *P* = 0.0001). Microvascular benefits included reduced retinopathy (OR: 0.83, 95% CI: 0.71–0.97, *P* = 0.02) and albuminuria (OR: 0.83, 95% CI: 0.70–0.99, *P* = 0.03). No significant differences were observed in all-cause mortality (OR: 0.86, 95% CI: 0.71–1.04, *P* = 0.12), CV mortality (OR: 0.77, 95% CI: 0.53–1.11, *P* = 0.16), or renal failure (OR: 0.96, 95% CI: 0.34–2.69, *P* = 0.94). Subgroup analysis showed diastolic BP-targeted trials had greater mortality reduction (OR: 0.52, 95% CI: 0.33–0.83, *P* = 0.005) versus systolic BP-targeted trials (*P* = 0.88). Serious adverse events did not differ between groups (OR: 1.28, 95% CI: 0.90–1.81, *P* = 0.17).

**Conclusion:**

In T2DM patients, IBPC reduces cardiovascular events and microvascular complications without increasing adverse events, supporting lower BP targets (<130/80 mm Hg). Diastolic BP control may offer additional mortality benefits.

## Introduction

Hypertension (HTN) is one of the leading causes of premature death, cardiovascular (CV) disease and all cause mortality^[^1^]^, responsible for approximately 7.5 million deaths worldwide^[^2^]^. One of the most common comorbidities associated with HTN is type 2 Diabetes mellitus (T2DM), which occurs due to shared risk factors such as obesity and lipid profile^[^3,4^]^. Despite the widespread impact of HTN, many older adults remain underdiagnosed or are inadequately treated^[^5^]^. Even if they get diagnosed, treatment and management of HTN is challenging due to its link with multiple comorbidities such as heart failure (HF), myocardial infarction (MI), and other cardiovascular disease^[^6^]^. Managing HTN in older adults T2DM often requires careful consideration of blood pressure control procedures, aiming to reduce the risk of cardiovascular events occurring while minimizing potential adverse effects. Despite the advancements in clinical guidelines, the optimal approach for blood pressure control in this population remains debated upon. Standard blood pressure control in patients with T2DM typically aims to achieve the target of systolic blood pressure (BP) ≤ 140 mm Hg and diastolic BP ≤ 90 mm Hg for older adults^[^7^]^. This approach helps in reducing the risk of cardiovascular or renal complications^[^8^]^. Standard treatment includes lifestyle modifications as well as pharmacological interventions. Research evidence proves that losing weight, more physical activities, reduced alcohol consumption and putting an end to smoking can have a significant impact on HTN in patients with T2DM^[^9^]^. But, changing your lifestyle options becomes increasingly hard for individuals in older age^[^10^]^. In the United States, when weight loss is recommended to older adults, increased physical activity is not always suggested^[^10^]^. Furthermore, angiotensin converting enzyme inhibitors (ACEIs) are generally considered as the first line treatment for diabetic hypertensive patients^[^11^]^. Other drugs which provide equal benefits, include diuretics, beta blockers, angiotensin II receptor blockers (ARBs), and calcium antagonists. However, the choice of drug is highly dependent on the patient’s specific condition, particularly the extent and severity of T2DM and HTN^[^11^]^. In contrast, T2DM leads to a 2- to 3-fold increase in the risk of cardiovascular events. Given the continuous and graded cardiovascular risk in patients with diabetic hypertension across the full range of levels of systolic blood pressure, including prehypertensive levels, it is recommended to begin intensive blood pressure control. The objective is to reduce the systolic blood pressure to below 130 mm Hg^[^12^]^. According to 2023 European Society of Hypertension (ESH) and 2017 American College of Cardiology/American Heart Association (ACC/AHA) hypertension guidelines, lower blood pressure (BP) target should be <130/80 mm Hg for older adults to significantly reduce the risk of CV events^[^13^]^. Additionally, older adults are more likely to experience reduced physiological and psychological capacities, higher risk of undergoing stress, and end organ damage due to diabetic hypertension^[^14^]^. These factors further contribute to the need for intensive blood pressure control in older adults to help mitigate the risks associated with diabetic hypertension.

Given the Standard blood pressure control’s limitation and the intensive blood pressure control’s emerging potential, a comparative analysis for older adults undergoing diabetic hypertension is essential. By pooling the currently available literature, we aim to offer an in-depth analysis of key clinical outcomes.

This study complies with the TITAN Guidelines 2025 on the declaration and use of artificial intelligence in research^[^15,16^]^.

## Methods

### Study design and protocol registration

The meta-analysis was completed according to the guidelines set forth in the preferred reporting items for systematic reviews and Meta-analysis (PRISMA) statement^[^17^]^, as well as the protocols outlined in the Cochrane Handbook for systematic reviews and meta-analyses^[^18^]^. This work has been reported in accordance with the AMSTAR (Assessing the Methodological Quality of Systematic Reviews) guidelines.

### Search strategy and databases

A comprehensive search of PubMed, Web of Science, and Embase was conducted from database inception through March 2025. We imposed no language or date restrictions during the search. The search strategy used the keywords “Diabetes Mellitus, Type 2” and “Antihypertensive Agents,” along with related terms. The complete search strings and results for each database are provided in (Supplemental Digital Content Table 1, available at: http://links.lww.com/MS9/B108). In addition, we manually screened the reference lists of all included studies to identify any further eligible publications.

**HIGHLIGHTS**

- IBPC cuts CV events in T2DM (OR: 0.82, *P* = 0.0001).
- Lowers stroke risk by 38% (OR: 0.62, *P* = 0.0001).
- Reduces retinopathy (OR: 0.83, *P* = 0.02).
- No mortality benefit (OR: 0.86, *P* = 0.12).
- SBP targets outperform SBP + DBP for CV outcomes.
- Grade: moderate evidence for microvascular benefits.

### Eligibility criteria

We included randomized controlled trials (RCTs) that enrolled adult patients (aged 35 years or older) with a confirmed diagnosis of type 2 diabetes mellitus (T2DM) and a baseline blood pressure greater than 110/70 mm Hg. Studies were eligible if they compared intensive blood pressure control (IBPC), typically involving lower blood pressure targets, with standard blood pressure control. Trials that targeted systolic blood pressure alone, diastolic blood pressure alone, or both were all considered eligible, as long as they compared an intensive versus standard approach. To be included, studies had to report at least one of the following outcomes: all-cause mortality, cardiovascular mortality, major cardiovascular events (such as myocardial infarction or stroke), cerebrovascular events, onset or progression of chronic kidney disease (CKD), incident heart failure, development of microalbuminuria or albuminuria, renal failure, diabetic retinopathy, diabetic neuropathy, or serious adverse events. Only RCTs were included to ensure methodological rigor, and although high-quality cohort studies were initially considered, they were ultimately excluded to maintain consistency. We excluded studies that did not compare intensive versus standard blood pressure control, as well as case reports, review articles, meta-analyses, editorials, conference abstracts, animal studies, and duplicate publications or secondary analyses of previously published data.

### Study selection

**Figure 1.**
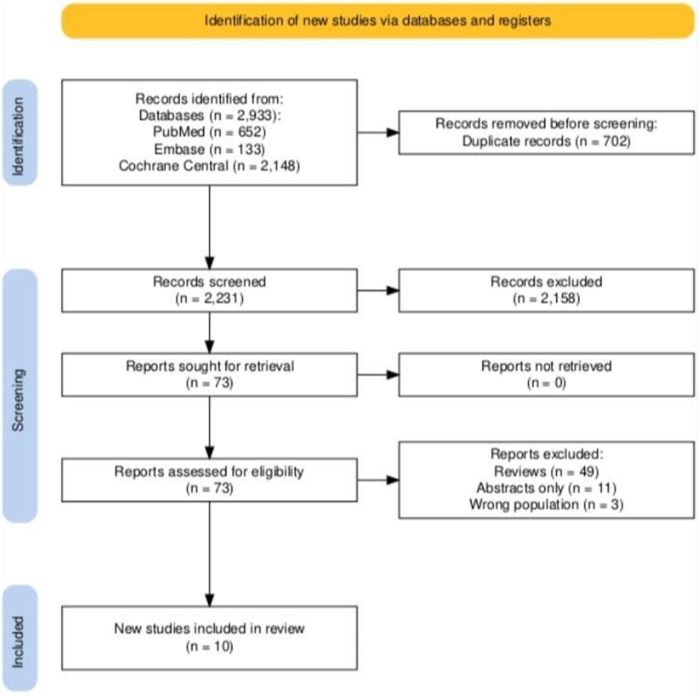
PRISMA flowchart of the study screening and selection process.

The articles from all three databases were imported to Rayyan software for screening. Duplicate studies were removed automatically by the software. Two authors S.R. and M.S. independently reviewed the articles based on title and abstract. Subsequently a comprehensive review of the full text of selected articles was conducted, based on our established inclusion criteria. Any discrepancies were addressed by the lead author. The details of the selection process are illustrated in the PRISMA flowchart (Fig. 1).

### Data extraction

We conducted a preliminary extraction after obtaining the full texts from the relevant studies. This was done to create an extraction template in Excel (Microsoft, U.S.A.), which was in three sections. In the study characteristics, first author’s name, year of publication, inclusion criteria, target blood pressure and follow up; whereas baseline characteristics included study ID, mean age, mean BP at baseline in IBPC and standard control group; and mean BP at follow up in IBPC group and standard blood pressure control group.

The primary outcomes included a composite of the first occurrence of nonfatal stroke, nonfatal myo-cardial infarction, treatment or hospitalization for heart failure, or death from cardiovascular causes; whereas secondary outcomes included All cause mortality, CVD related deaths, cardiovascular events, events of any stroke and cerebrovascular event, onset or progression of CKD, incidence of heart failure, Incidence of microalbuminuria or incident albuminuria, renal failure, retinopathy, neuropathy, and serious adverse events. Three authors Z.B., S.R., and M.S. extracted the data from our selected studies using this criterion as the standard form in an Excel sheet. The first author addressed and resolved all conflicts.

### Quality assessment and risk of bias

To assess the risk of bias in our study, we employed the revised Cochrane Collaboration tool for RCTs (ROB 2)^[^19^]^ for all the randomized control trials. The evaluation indicators included randomization sequence generation, allocation concealment, blinding, incomplete outcome data, and selective reporting (Supplemental Digital Content Figure 1, available at: http://links.lww.com/MS9/B108).

#### Grade assessment

The quality of the evidence for this meta-analysis was assessed by two independent reviewers (AK and MAA) using the GRADE framework, with the GRADEpro Guideline Development Tool^[^20^]^. The evidence was classified into levels ranging from high to very low^[^21^]^. Any discrepancies in the assessment were resolved through mutual agreement.

### Statistical analysis

Statistical analysis was performed using Review Manager [(RevMan) [Computer program] Version 5.4. The Cochrane Collaboration, 2020]. A fixed-effects model was used unless the pooled studies showed considerable heterogeneity (*I*^2^ > 50%), at which point the random-effects model was used instead^[^22^]^. All the dichotomous outcomes were reported as Odds Ratios (OR) with a 95% confidence interval (CI). We employed the chi-square test and the *I*^2^ statistic to evaluate and quantify the level of heterogeneity in the data. A *P*-value of less than 0.1 from the chi-square test indicated significant heterogeneity. When *I*^2^ was greater than 50%, a sensitivity test was conducted to address it. An overall *P*-value of less than 0.05 was regarded as statistically significant^[^23^]^.

#### Subgroup analysis

To explore the differential impact of IBPC based on targeted blood pressure components, we conducted a predefined subgroup analysis. Trials were categorized into three groups according to their BP targets: systolic BP (SBP) only, diastolic BP (DBP) only, and both SBP + DBP. Outcomes included in the subgroup analysis were those reported by at least two trials within each subgroup to maintain statistical validity.

## Results

### Search results

A total of 2933 records were identified through database searches: 652 from PubMed, 133 from Embase, and 2148 from Cochrane Central. After removing 702 duplicate records, 2231 records remained for title and abstract screening. Of these, 2158 records were excluded based on relevance, and 73 full-text articles were assessed for eligibility. No reports were excluded due to retrieval issues. Among the 73 full-text articles assessed, 63 were excluded for the following reasons: 49 were review articles, 11 were available as abstracts only, and 3 included the wrong population. Ultimately, 10 studies met the eligibility criteria and were included in the final meta-analysis (Fig. 1).

### Study characteristics

A total of 10 randomized controlled trials^[^24–32^]^ were included in this meta-analysis, comprising patients with type 2 diabetes and varying blood pressure targets. The total sample size included 23 826 participants.The studies were published in China,Sweden, Italy, Canada, US, and UK. Publication years ranged from 1998 to 2025 (Table 1). Table 2 provides the summary of baseline characteristics for included studies.

**Table 1:** Study characteristics.

Study ID	Year	Country	Intervention	Control	median follow up, years	Outcomes reporting
ACCORD	2010	US	IBPC	Standard control	4.7	Primary composite outcome, All-cause mortality, any stroke/cerebrovascular event, CVD deaths, CVD events, heart failure, microalbuminuria/incidenturia, renal failure, serious adverse events
HOT	1998	Sweden, Italy, Canada	IBPC	Standard control	3.8	All-cause mortality, any stroke/cerebrovascular event, CVD deaths, CVD events
UKPDS-38	1998	UK	IBPC	Standard control	8.4	All-cause mortality, any stroke/cerebrovascular event, CVD events, heart failure, renal failure
ABCD	2000	US	IBPC	Standard control	5.3	All-cause mortality, CVD events, neuropathy, retinopathy
ABCD-2V	2006	US	IBPC	Standard control	1.9	CVD deaths, CVD events, microalbuminuria/incidenturia
J-DOIT3	2017	Japan	IBPC	Standard control	8.5	Primary composite outcome, All-cause mortality, any stroke/cerebrovascular event, CKD onset /progression /nephropathy, CVD deaths, CVD events, heart failure, retinopathy, serious adverse events
SANDS	2008	US	IBPC	Standard control	3	Primary composite outcome, any stroke/cerebrovascular event, CVD deaths, serious adverse events
Shi *et al* trial	2020	China	IBPC	Standard control	7	Microalbuminuria/incidenturia
ADDITION Leicester	2020	UK	IBPC	Standard control	5	Primary composite outcome, All-cause mortality, CKD onset /progression /nephropathy, CVD events, heart failure, neuropathy, retinopathy
Bi *et al* trial	2025	China	IBPC	Standard control	4.2	Primary composite outcome, All-cause mortality, any stroke/cerebrovascular event, CKD onset /progression /nephropathy, CVD deaths, CVD events, heart failure, microalbuminuria/incidenturia, renal failure, serious adverse events

**Table 2:** Baseline characteristics of included studies.

Study ID	Target blood pressure (mm Hg)	No. participants with diabetes	Mean age (years)	Men (%)	Mean BP baseline (intensive BP group)	Mean BP baseline (standard BP group)	Mean BP follow-up (intensive BP group)	Mean BP follow-up (standard BP group)
ACCORD	Systolic blood pressure <120 vs < 140	2362/2371	62	52	139.0/75.9	139.4/76.0	119.3/64.4	133.5/70.5
HOT	Diastolic blood pressure <90 vs < 85 vs < 80	499/501	61.5	53	NR	NR	NR	NR
UKPDS-38	Blood pressure <150/ 85 vs < 180/105	758/390	56.5	56	159.0/94.0	160.0/94.0	144.0/82.0	154.0/87.0
ABCD	Diastolic blood pressure <75 vs < 80–89	237/233	57.9	67.5	156.0/98.0	154.0/98.0	132.0/78.0	138.0/86.0
ABCD-2V	Diastolic blood pressure <75 vs < 80–89	66/63	56.1	67.5	126.0/84.0	126.0/84.0	118.0/75.0	124.0/80.0
J-DOIT3	Blood pressure <120/ 75 vs < 130/80	1269/1271	59	62	133.5/79.3	134.1/80	124.9/71.3	128.2/72.9
SANDS	Blood pressure <115/ 75 vs < 130/85	252/247	56	34.5	128.0/74.0	133.0/76.0	117.0/67.0	129.0/73.0
Shi *et al* trial	Blood pressure <130/ 85 vs < 160/95	75/75	48.8	50	129.1/79.8	128.8/76.9	120.7/76.2	127.8/79.4
ADDITION Leicester	Blood pressure <130/ 80 vs < 140/85	144/192	59.5	57.8	145.8/88.0	148.3/89.7	126.9/73.7	139.2/80.9
Bi *et al* trial	Systolic blood pressure <120 vs < 140	6414/6407	63.8	54.7	140/76.3	140/76.3	121.6/69	133.2/74

### Risk of bias assessment

#### Quality assessment

Of the ten randomized controlled trials (RCTs) included, seven were assessed as having some concerns regarding risk of bias. Among these, five trials demonstrated concerns related to deviations from the intended interventions, one trial exhibited bias arising from the randomization process, and one trial showed both deviations from the intended intervention and bias due to missing outcome data. The remaining two trials were judged to have a low risk of bias across all domains. Notably, none of the studies were rated as having a high risk of bias. A detailed risk of bias assessment for each study is presented in Supplemental Digital Content Figure S1, available at: http://links.lww.com/MS9/B108.

#### Certainty of evidence

The GRADE approach, using the GRADE pro Guideline Development Tool, was employed to assess the certainty of evidence. A detailed assessment is shown in Supplemental Digital Content Table S2, available at: http://links.lww.com/MS9/B108.

### Primary outcome

**Figure 2.**
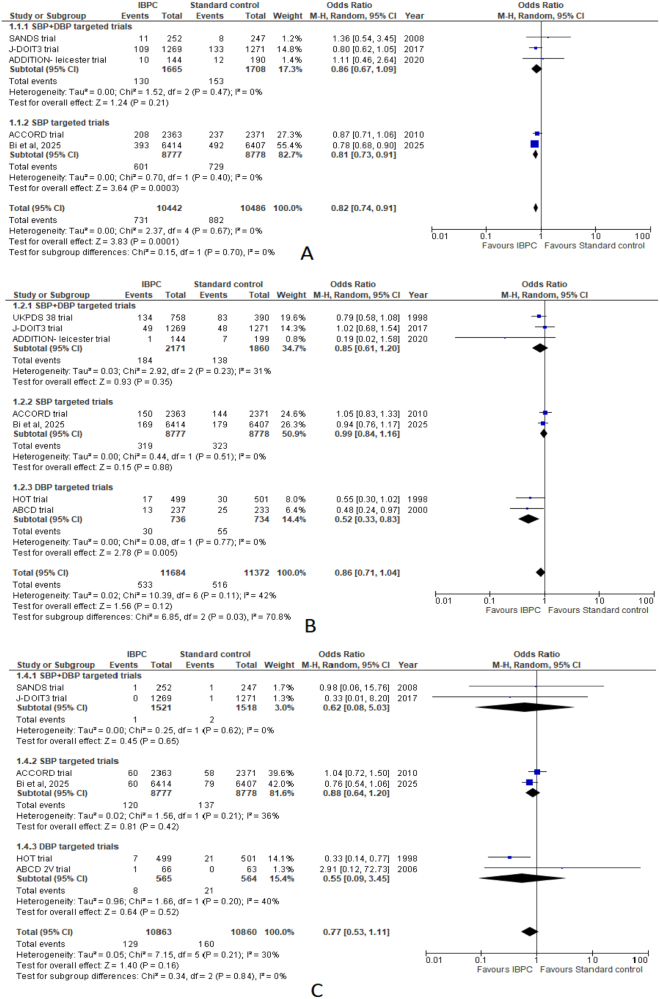
Forest plots comparing intensive blood pressure control (IBPC) with standard blood pressure control for: (A) primary composite outcome, (B) all-cause mortality, (C) cardiovascular disease (CVD)-related deaths.

Pooled analysis of five studies revealed statistically significant lower odds in the IBPC group (OR: 0.82; 95% CI: 0.74–0.91; *P* = 0.0001; *I*^2^ = 0%). Subgroup analysis noted statistically significant lower odds for SBP-targeted trials in the IBPC arm (OR: 0.81; 95% CI: 0.73–0.91; *P* = 0.003; *I*^2^ = 0%), whereas SBP + DBP targeted trials noted a slightly lower but insignificant odds of the primary outcome in the IBPC arm (OR: 0.86; 95% CI: 0.67–1.09; *P* = 0.21; *I*^2^ = 0%) (Fig. 2A).

### Meta-analysis of secondary outcomes

#### All-cause mortality

Pooled analysis of seven studies with 11 684 patients in the intervention arm and 11 372 patients in the control arm revealed no difference in all-cause mortality between both groups (OR: 0.86; 95% CI: 0.71–1.04; *P* = 0.12). The estimate displayed mild heterogeneity (*I*^2^ = 42%, *P* = 0.11).

Subgroup analysis was conducted based on SBP, DBP, and SBP + DBP targeted trials. DBP-targeted trials noted significantly lower odds of all-cause mortality in the IBPC arm (OR: 0.52; 95% CI: 0.33–0.83; *P* = 0.005; *I*^2^ = 0%). Both SBP (OR: 0.99; 95% CI: 0.84–1.76; *P* = 0.88; *I*^2^ = 0%) and SBP + DBP targeted trials (OR: 0.85; 95% CI: 0.61–1.20; *P* = 0.35; *I*^2^ = 31%) noted no significant difference between both groups (Fig. 2B).

#### CVD-related death

Analysis of six studies recorded a slightly lower but insignificant incidence of CVD-related deaths in the IBPC arm (OR: 0.77; 95% CI: 0.53–1.11; *P* = 0.16), with mild heterogeneity (*I*^2^ = 30%, *P* = 0.21).

Subgroup analysis noted slightly lower but insignificant odds of CVD-related death among DBP-targeted trials (OR: 0.55; 95% CI: 0.09–3.45; *P* = 0.52; *I*^2^ = 40%). Among SBP + DBP targeted trials, a reduced but insignificant incidence of CVD-related deaths was noted (OR: 0.62; 95% CI: 0.08–5.03; *P* = 0.65; *I*^2^ = 0%), while SBP targeted trials noted similar conclusions (OR: 0.88; 95% CI: 0.64–1.20; *I*^2^ = 36%) (Fig. 2C).

#### Cardiovascular events

The pooled estimate of nine studies revealed lower odds of cardiovascular events in the IBPC arm (OR: 0.84; 95% CI: 0.76–0.94; *P* = 0.002), with no heterogeneity (*I*^2^ = 0%).

**Figure 3.**
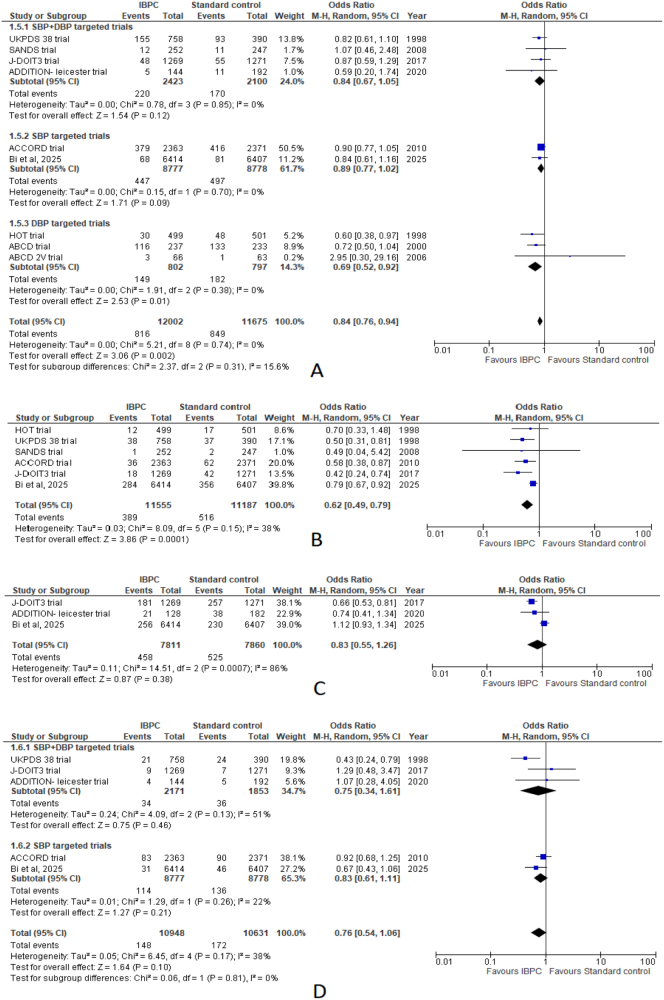
Forest plots comparing IBPC with standard blood pressure control for: (A) cardiovascular events, (B) any stroke or cerebrovascular events, (C) chronic kidney disease (CKD) onset, progression, or nephropathy, (D) heart failure.

Subgroup analysis revealed significantly lower odds of CVD events among DBP-targeted trials (OR: 0.69; 95% CI: 0.52–0.92; *P* = 0.01; *I*^2^ = 0%). However, both SBP-targeted trials (OR: 0.89; 95% CI: 0.77–1.02; *P* = 0.09; *I*^2^ = 0%) and SBP + DBP-targeted trials (OR: 0.84; 95% CI: 0.67–1.05; *P* = 0.12; *I*^2^ = 0%) noted insignificant differences between both groups (Fig. 3A).

#### Event of any stroke or cerebrovascular event

The pooled estimate of six studies revealed significantly lower incidence of stroke or cerebrovascular events in the IBPC arm (OR: 0.62; 95% CI: 0.49–0.79; *P* = 0.0001). Across the outcome, studies noted mild heterogeneity (*I*^2^ = 38%, *P* = 0.15) (Fig. 3B).

#### Onset or progression of CKD

Analysis of three studies revealed lower but insignificant odds of CKD onset or progression in the IBPC arm (OR: 0.83; 95% CI: 0.55–1.26; *P* = 0.38) with high heterogeneity (*I*^2^ = 86%, *P* = 0.0007) (Fig. 3C). Heterogeneity was resolved following sensitivity analysis and exclusion of Bi *et al* due to its disproportionately large sample size compared to the other two studies reporting this outcome, to assess its influence on the pooled effect, with effect size favoring the IBPC arm (OR: 0.67; 95% CI: 0.55–0.81; *P* < 0.0001) (Supplemental Digital Content Figure 2A, available at: http://links.lww.com/MS9/B108).

#### Incidence of heart failure

On a pooled estimate of five studies, IBPC noted a slightly reduced but insignificant incidence of heart failure (OR: 0.76; 95% CI: 0.54–1.06; *P* = 0.10), with low heterogeneity (*I*^2^ = 38%, *P* = 0.17) (Fig. 3D).

#### Incidence of microalbuminuria or incident albuminuria

**Figure 4.**
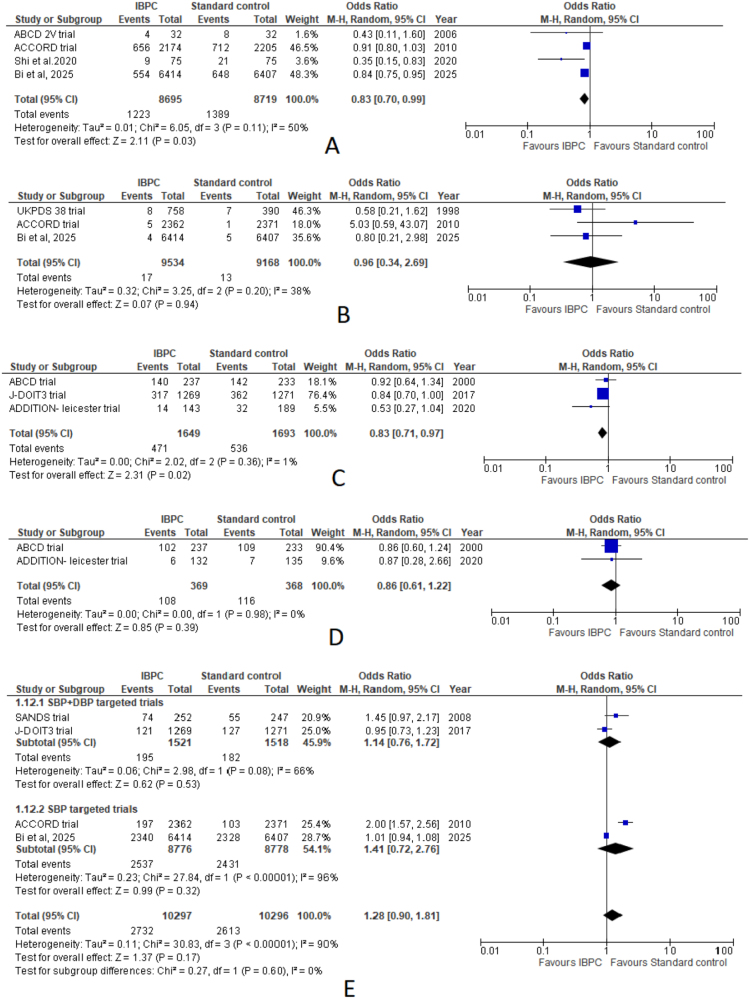
Forest plots comparing IBPC with standard blood pressure control for: (A) incidence of microalbuminuria or albuminuria, (B) renal failure, (C) retinopathy, (D) neuropathy, (E) serious adverse events.

The pooled results of four studies revealed lower odds of microalbuminuria or albuminuria in the IBPC arm (OR: 0.83; 95% CI: 0.70–0.99; *P* = 0.03) with moderate heterogeneity (*I*^2^ = 50%, *P* = 0.11) (Fig. 4A).

#### Renal failure

Similar odds of renal failure were noted on pooled analysis of three studies between both arms (OR: 0.96; 95% CI: 0.34–2.69; *P* = 0.94). The pooled estimate noted low heterogeneity (*I*^2^ = 38%, *P* = 0.20) (Fig. 4B).

#### Retinopathy

The pooled estimate of three studies revealed statistically significant odds of retinopathy in the IBPC arm (OR: 0.83; 95% CI: 0.71–0.97; *P* = 0.02). Pooled analysis noted low heterogeneity (*I*^2^ = 1%, *P* = 0.36) (Fig. 4C).

#### Neuropathy

The pooled estimate of two studies revealed slightly lower yet insignificant odds of neuropathy in the IBPC arm (OR: 0.86; 95% CI: 0.61–1.22; *P* = 0.39), with no heterogeneity (*I*^2^ = 0%, *P* = 0.98) (Fig. 4D).

#### Serious AE

The pooled estimate of four studies noted insignificant odds of serious AE in the IBPC arm (OR: 1.28; 95% CI: 0.90–1.81; *P* = 0.17). Pooled estimate noted high heterogeneity (*I*^2^ = 90%, *P* < 0.0001) (Fig. 4E), which was brought to low (*I*^2^ = 40%, *P* = 0.19) following the exclusion of ACCORD trial, with similar findings (OR: 1.04; 95% CI: 0.89–1.22; *P* = 0.63) (Supplemental Digital Content Figure 2B, available at: http://links.lww.com/MS9/B108).

## Discussion

This meta-analysis evaluated the impact of intensive blood pressure control (IBPC) compared to standard care in patients with type 2 diabetes mellitus (T2DM) across a broad spectrum of cardiovascular, renal, and microvascular outcomes. The primary outcome – a composite of the first occurrence of nonfatal stroke, nonfatal myocardial infarction, treatment or hospitalization for heart failure, or death from cardiovascular causes – was significantly reduced in the IBPC group. Additionally, IBPC was associated with lower odds of cardiovascular events, stroke, microalbuminuria, and retinopathy. However, no statistically significant differences were observed in cardiovascular mortality or heart failure outcomes. These findings are partially aligned with those of Adil *et al*, a retrospective observational study that investigated the impact of intensive systolic blood pressure (SBP) control targeting <120 mm Hg in patients with T2DM^[^15^]^. Their results demonstrated that more intensive SBP control was associated with a significant reduction in major cardiovascular events, including myocardial infarction, stroke, and cardiovascular death, but also reported an increased incidence of adverse effects such as hyperkalemia and symptomatic hypotension^[^15^]^. Although relevant, this study was excluded from our analysis due to its retrospective design.

These findings align with a meta-analysis by Yang *et al*, which included trials with achieved SBP levels ranging from 117 to 144 mm Hg. The lowest risk of major cardiovascular disease (CVD) was observed at SBP levels of 120–124 mm Hg, with hazard ratios of 0.73 (vs 130–134 mm Hg), 0.60 (vs 140–144 mm Hg), and 0.41 (vs ≥150 mm Hg). Similar reductions were noted in stroke, myocardial infarction, heart failure, and cardiovascular death. Although all-cause mortality decreased at an SBP level of <140 mm Hg, further SBP reduction offered no added survival benefit^[^33^]^. In contrast, our meta-analysis did not show a significant reduction in all-cause mortality overall; however, subgroup analysis revealed a significant mortality benefit in trials targeting DBP alone. No such benefit was observed in SBP-targeted or SBP + DBP-targeted trials. Differences in BP target strategies across studies may explain these discrepancies. Notably, the ACCORD^[^24^]^ and^[^32^]^ trials, which targeted SBP levels of <120 mm Hg in the intervention group, closely align with the SBP range associated with the lowest CVD risk in Yang *et al*’s analysis.

Our study extends the findings of McBrien *et al*, who analyzed five RCTs involving 7312 type 2 diabetes patients and found that intensive blood pressure control had a relatively modest benefit on macrovascular outcomes, with a higher number needed to treat (NNT) to prevent one death, myocardial infarction, or stroke compared to standard control. However, their analysis did not assess key microvascular or safety outcomes such as serious adverse events (SAEs), retinopathy, neuropathy, or albuminuria^[^34^]^. Our meta-analysis addressed this gap by evaluating these outcomes, finding no significant difference in SAEs or neuropathy, but a significant reduction in retinopathy and microalbuminuria in the IBPC group – highlighting potential long-term microvascular benefits, especially in early diabetic organ damage. Additionally, unlike McBrien *et al*, who did not pool data on heart failure^[^34^]^, we included it in our analysis and found a slightly reduced but statistically insignificant risk with IBPC. These findings emphasize that while macrovascular benefits may be limited, incorporating microvascular and underreported outcomes provides a more complete and individualized picture of the risks and benefits of intensive BP control in type 2 diabetes.

A recent meta-analysis by Ioannidou *et al* assessed renal outcomes – including ESRD, renal failure, and CKD progression/onset – in patients with type 2 diabetes undergoing intensive versus standard BP control. Their findings showed no statistically significant differences between groups and were limited by high heterogeneity, which may have affected the accuracy of results^[^35^]^. Our meta-analysis provided a more detailed evaluation by analyzing renal failure, CKD progression/onset, and nephropathy (microalbuminuria/albuminuria) as separate outcomes. Initially, these outcomes showed no significant difference and considerable heterogeneity. However, a sensitivity analysis excluding the study by Bi *et al*^[^32^]^ resolved the heterogeneity and revealed a statistically significant benefit in the IBPC arm. This improvement is likely due to differences in trial design – Bi *et al*^[^32^]^ focused exclusively on SBP targets, while trials like ADDITION-Leicester^[^36^]^ and J-DOIT3^[^29^]^ used combined SBP and DBP targets. Although we did not perform BP-targeted subgroup analysis for renal outcomes specifically, these findings underscore the importance of uniform BP target definitions and suggest that differing BP strategies across studies can significantly influence pooled renal outcome results.

In addition to assessing individual outcomes, our study performed a detailed subgroup analysis based on BP targets – SBP, DBP, and SBP + DBP – focusing only on outcomes reported by at least two studies per subgroup. For the primary composite outcome, a significant reduction was observed in SBP-targeted trials, while SBP + DBP trials showed no significant effect, suggesting that focused SBP control may be more effective in reducing macrovascular risk. All-cause mortality and cardiovascular events both showed significant reductions only in DBP-targeted trials, while SBP and SBP + DBP subgroups did not demonstrate significant effects. For cardiovascular mortality, no subgroup showed a statistically significant benefit, although DBP-targeted trials showed a trend toward benefit in cardiovascular events, not mortality. This indicates that DBP control may be more influential in reducing overall cardiovascular burden rather than cardiovascular death specifically in patients with type 2 diabetes. No significant differences were found for heart failure or serious adverse events across SBP or SBP + DBP subgroups, suggesting that the type of BP target may not substantially affect these particular outcomes. Overall, these findings imply that the benefits of intensive blood pressure control vary depending on the targeted parameter – DBP-focused control may be more effective in reducing all-cause mortality and cardiovascular events, whereas SBP targeting seems more beneficial for composite outcomes. These insights support the need for individualized blood pressure goals, tailored to each patient’s cardiovascular and renal risk profile.

## Strength and limitations

This meta-analysis has several notable strengths. Firstly, it exclusively included randomized controlled trials, ensuring a high level of evidence with minimized risk of bias. We conducted a comprehensive assessment of both macrovascular and microvascular outcomes, including cardiovascular events, stroke, heart failure, CKD progression, retinopathy, neuropathy, and serious adverse events – some of which had not been thoroughly analyzed in prior reviews. Another strength lies in our detailed subgroup analysis based on SBP-, DBP-, and SBP + DBP-targeted trials, which provided valuable insight into how the type of blood pressure target may influence outcomes. For example, DBP-targeted trials consistently showed significant benefits of intensive blood pressure control on all-cause mortality and cardiovascular events. Moreover, we addressed heterogeneity through sensitivity analyses – most notably in the CKD progression outcome, where exclusion of the Bi *et al*^[^32^]^ study resolved heterogeneity and revealed a significant benefit in the intensive group. The inclusion of recent trials like^[^32^]^ also makes our analysis more contemporary and reflective of current practice.

However, our study is not without limitations. The included trials varied in their target BP thresholds, duration of follow-up, and definitions of outcomes, which may have contributed to heterogeneity in effect sizes. Some outcomes, such as neuropathy, retinopathy, and serious adverse events, were assessed in only a limited number of studies, reducing the power to detect differences and limiting the generalizability of those findings. While our decision to include only RCTs enhances methodological rigor, it also meant excluding valuable data from large-scale observational studies which showed significant cardiovascular benefit but were not eligible for inclusion. Additionally, variability in follow-up durations across studies may have influenced the detection of long-term complications such as renal failure or neuropathy. Lastly, despite our efforts, the potential for publication bias remains, especially for outcomes that may not be reported when findings are negative or non-significant.

## Data Availability

Data supporting the findings of this study are available from the corresponding author upon reasonable request.

## Research registration unique identifying number (UIN)

Registry: PROSPERO. Registration ID: CRD420251036677. Registration link: https://www.crd.york.ac.uk/prospero/display_record.php?ID=CRD420251036677.

## Permission to reproduce material from other sources

No copyrighted material from other sources was reproduced in this article.
